# Need of More Medical Officers

**Published:** 1918-03

**Authors:** 


// BUFFALO MEDICAL JOURNAL

**A Monthly Review of Medicine and Surgery**

EDITOR AND PUBLISHER, DR. A. L. BENEDICT, 228 Summer St., corner of Elmwood Ave., Buffalo, (Address for all communications. Please make personal and telephone calls before 1 P. M.)

Third, in age, on the western continent. Independent, but supports professional organizations. News limited mainly to Western N. Y. and Penn.

Subscription $2.00 a year; $3.00 for two years in advance. Changes and errors in address or failure or duplication of delivery, should be reported immediately.

Yearly Volume 73 MARCH, 1918 Number 8

### Need of More Medical Officers.

In the spring, it was stated that about 20,000 physicians would be needed for the U. S. army. Rather less than this number has been raised but, with the considerably increased regular army medical corps and the recall of many retired—and not necessarily superannuated or incapacitated—surgeons, and the surgeons taken over with the national guard, just about the requisite number has been secured for the present and the immediate future as represented by the demands of the first and second draft calls. Largely to make good the shortage of surgeons among our allies, it is now stated that about 15,000 more will be needed, so that the remarks to follow are still in order.

Passing over the possibility of an early cessation of hostilities, in regard to which hindsight will be more valuable than foresight, it should be thoroughly comprehended that the war cannot be fought by those who have nothing better to do, who will sacrifice little beyond personal convenience and hazard, or who have not urgent private business and responsibilities to dependents. If anything, this is more true of the medical men than of the strictly combatant forces. When we realize that the average physician is 26 at graduation and 27 at the completion of his interne service, and that the number of graduates has fallen off in the last 12 years to approximately 60% of what it was for the same preceding period, it is evident that the medical personnel simply cannot be secured even by a draft of every physician of an age corresponding to that for which we at present expect to draft about 20% of the male population generally. Accurate statistics are not at hand but, so far as can be determined by observation of the medical personnel of a division (i.e. about 250 physicians in a mass of 25,000 men) about 1/3 are in the neighborhood of 30, 1/3 near 50, and 1/3 between these extremes. It is no time for the physician to feel that grey hair or little hair at all exempts him from the duty of volunteering. It is no time to wait for a medical draft, however desirable that may be for the rank and file of the army. No draft on the medical profession can operate with as little hardship as the sincere personal judgment of the individual physician, as to the actual need of his services to his clientele or hospital on the one hand and to his country as an organized fighting force on the other.

The same general fact can be better stated in a still more personal way. Disregard the matter of relative duty to private and to military demands. Disregard the problem of what you will do when the war is over. If it is short, your practice will return to you even more surely than if you took a fairly long vacation or a post graduate course—and from many aspects, medical service is both. If the war is long, there will be such a readjustment of supply and demand, that any fairly well qualified physician can get a practice anywhere or, what is still more plausible, the medical profession will be "socialized" in one way or another, and both employment and living income will be assured. Don't imagine because you have got into middle-aged ways of thinking of yourself and are accustomed to certain luxuries and conveniences, that you cannot stand the hardships of a healthy outdoor life. If the matter of personal hazard occurs to you at all, the best statistics available show that, for a very young man, the strictly military risk of being killed or injured to a serious degree is at most, double that of death and physical injury in civil life, with a slight reduction of the non-military risks on account of better hygiene and sanitation and discipline. For a man close to the maximum age for service, with a due allowance for his probable utilization in the rear, the strictly military risk is both relatively and actually very small, probably not much if any greater than that directly due to the possession and use of an automobile. If he is organically diseased or really in a state of presenility, the hardships of military life will probably result in early incapacity and retirement, or in a considerable increase in probability of death from indirect causes, but the opportunity to live more actively and to acquire a new experience compensate for the actual average shortening of life, not to mention the fact that military service will give many men an extension of their lease on life.

Don't "weigh" the advantages and duties of civil and military practice. Put it to yourself in this way: "Can I or can I not volunteer for military service without seriously affecting my ability to support my family or without sacrificing whatever capital I have accumulated for future use?" Any man who has to consider himself alone, can live well on even a lieutenant's salary and perquisites. Moreover, there have been no estimates of average medical earnings for two generations that show a net income approaching this amount. But don't volunteer unless you are willing to accept active service when you are called and to take what comes.

